# Blended-learning with half the face-to-face time versus conventional abdominal ultrasound training in undergraduate medical education: a randomized controlled non-inferiority trial

**DOI:** 10.1186/s12909-026-08914-4

**Published:** 2026-02-28

**Authors:** Laura Butennandt, Tina Stibane, Andreas Mayr, Felix Mühlensiepen, Helmut Sitter, Johannes Knitza

**Affiliations:** 1 Dr. Reinfried Pohl Center for Medical Education, Philipps-Universität Marburg, Marburg, Germany; 2 Institute for Digital Medicine, Philipps-Universität Marburg, Baldingerstrasse 1, Marburg, 35043 Germany; 3 Institute for Medical Biometry and Statistics, Philipps-Universität Marburg, Marburg, Germany; 4 Center for Health Services Research, Faculty of Health Sciences, Brandenburg Medical School Theodor Fontane, Rüdersdorf, Germany

**Keywords:** Blended learning, Abdominal ultrasound training, Undergraduate medical education, Competency-based training, Clinical skills acquisition

## Abstract

**Background:**

Ultrasonography is an essential clinical tool, offering rapid, bedside, imaging that supports timely clinical decision-making. Its effectiveness, however, depends heavily on examiner skill, requiring structured, practice-oriented training. Traditional tutor-led ultrasound teaching is limited by personnel and resource shortages. Blended learning, combining self-directed digital preparation with targeted hands-on teaching, may help overcome these constraints while maintaining educational quality. This randomized controlled non-inferiority trial evaluated whether a blended-learning abdomen ultrasound curriculum with reduced face-to-face teaching is as effective as a conventional full-day peer-teaching course.

**Methods:**

Clinical-phase medical students at Philipps University Marburg were eligible to participate. After enrollment and written informed consent, students were randomized to either a conventional one-day (8-hour) face-to-face curriculum or a blended-learning curriculum consisting of online preparatory modules, followed by a shortened, 4-hour hands-on session. Both formats were delivered by trained student peer tutors. The primary outcome was performance in an Objective Structured Clinical Examination (OSCE; 0–20 points). Secondary outcomes included theoretical knowledge assessed through a written test (0–20 points) and student perceptions measured via a Likert-scale questionnaire.

**Results:**

A total of 118 students were randomized (60 blended learning; 58 conventional). Blended learning was non-inferior to the conventional curriculum in OSCE performance (14.68 ± 2.72 vs. 14.86 ± 2.67; mean difference 0.18; 95% CI − 0.80 to 1.16), with the confidence interval fully within the non-inferiority margin of Δ = 4. Mean theoretical knowledge scores did not differ significantly (16.35 ± 2.74 vs. 16.59 ± 2.69; *p* = 0.35). Student evaluations indicated high perceived knowledge and skills gain in both groups, with comparable acceptance and course organization ratings.

**Conclusions:**

A blended-learning curriculum that shifts theoretical instruction from face-to-face teaching to structured self-directed online preparation, was non-inferior to a conventional full-day ultrasound course in teaching practical abdominal ultrasound skills. This approach provides an effective and resource-efficient strategy to broaden access to ultrasound education while preserving instructional quality.

**Trial registration:**

Not applicable.

**Supplementary Information:**

The online version contains supplementary material available at 10.1186/s12909-026-08914-4.

## Introduction

Ultrasonography is clinically vital because it delivers rapid, bedside, real-time information that supports quick diagnoses, immediate clinical decisions, and objective monitoring of disease and treatment [1]. Its radiation-free, non-invasive, portable, and cost-effective nature makes it usable across settings, from outpatient clinics to intensive care [2, 3]. Ongoing technical advances, including Doppler and contrast-enhanced techniques, have further increased image quality and diagnostic precision, cementing ultrasound as a core tool in most medical specialties [4].

At the same time, ultrasound remains strongly operator-dependent [5, 6]. The clinical value of an examination hinges on the examiner’s ability to acquire high-quality images, interpret findings correctly, and report them in a structured way. These competencies require substantial hands-on practice, anatomical understanding, and pattern-recognition skills, meaning that longitudinal, skills-focused education is essential from undergraduate training through residency [7, 8].

A major barrier to building these competencies is the shortage of qualified teaching staff and learning opportunities [4, 9]. Many faculties and training centers cannot meet demand with traditional tutor-led formats alone. To compensate, various strategies have been explored, including peer-teaching models [10] and approaches that shift parts of instruction to learners themselves, such as self-directed or technology-supported preparation. At Philipps University of Marburg, extracurricular full-day ultrasound courses at the Dr. Reinfried Pohl Center for Medical Education (Maris) follow a peer-to-peer model, which has shown positive results in ultrasound training [11–13]. Rising enrollment to the courses is increasingly constrained by limited tutor availability, room capacity, and equipment.

Blended learning offers a promising way to address these limitations by pairing self-directed digital preparation with targeted face-to-face scanning practice [14]. Hybrid formats, widely adopted during the pandemic, can improve flexibility, use resources more efficiently, and support learning [15, 16]. Importantly, surveys revealed that both instructors [17] and medical students [18] clearly preferred a blended-learning format for ultrasound education. This study therefore evaluated whether a blended learning concept with lower human resource involvement can expand access to ultrasound courses while maintaining high educational quality.

## Methods

### Study design

A prospective, randomized controlled parallel group non-inferiority trial was conducted during the winter term 2022/2023. On-site teaching took place at the Dr. Reinfried Pohl Center for Medical Education, Philipps University Marburg. The course was offered as a voluntary extracurricular activity, and all participants provided written informed consent before enrollment. The trial was approved by the Ethics Committee of University Hospital Marburg, Germany (reference number 118/22). The study was conducted and reported in accordance with the Consolidated Standards of Reporting Trials (CONSORT) guidelines for randomized clinical trials and the extension statement for non-inferiority trials.

### Population

All medical students at Marburg University who had passed the first German state examination and were therefore in the clinical phase of training were eligible to participate. Students were notified of the course offering through an institutional email distribution list and invited to enroll in the study. Students received no financial incentives.

### Randomization

After course registration and provision of written informed consent, participants were randomized to either the blended-learning curriculum or the conventional face-to-face curriculum. Randomization was based on a 1:1 group allocation ratio. Due to the nature of the intervention, neither instructors nor participants were blinded to group assignment.

### Traditional face-to-face curriculum

Following the recommendations of the German Society for Ultrasound in Medicine (DEGUM) [19], we developed a standardized set of ultrasound views and sections (Table 1). The extracurricular course was delivered as a one-day (8-hour) peer-to-peer program, alternating between focused theoretical teaching and supervised hands-on practice. Special attention was given to the FAST-protocol [20] to ensure all students are able to perform this emergency medicine procedure.

**Table 1:** Selected standard sections, structures and common pathologies to detect

Structure / protocol	Ultrasound standard sections according to DEGUM	Common pathologies to detect
Liver	⋅ Transverse scan along the midline ⋅ Longitudinal scan of the midline ⋅ Oblique subcostal scan ⋅ Sagittal scan	⋅ Inhomogeneous parenchyma ⋅ Inhomogeneous liver surface ⋅ Dilated liver veins ⋅ Hepatic steatosis
Gallbladder and bile ducts	⋅ Oblique subcostal scan ⋅ Shoulder-navel-section	⋅ Acute cholecystitis
Kidneys	⋅ Longitudinal section ⋅ Transverse section	⋅ Cysts ⋅ Hydronephrosis
Abdominal aorta	⋅ Transverse scan along the midline ⋅ Longitudinal scan of the midline	⋅ Aneurysm ⋅ Atherosclerotic plaque
Vena cava	⋅ Sagittal scan	⋅ Dilated vena cava
Spleen	⋅ Longitudinal section ⋅ Transverse section	⋅ Splenomegaly
Pancreas	⋅ Transverse scan along the midline ⋅ Longitudinal scan of the midline	-
FAST-protocol	⋅ Sub xiphoidal (pericardium) ⋅ Sagittal scan right (Morrison-Pouch) ⋅ Sagittal scan left (Koller-Pouch) ⋅ Suprapubic transverse scan (pouch of Douglas / rectovesical pouch)	⋅ Free fluid

### Blended learning curriculum

Building on the selected structures (Table 1) and the teaching materials used in the conventional course, we created self-directed preparatory modules specifically for this study. Each module began with a brief anatomical review and then provided a structured scanning guide, normal findings, and common pathologies. All materials were hosted on the AMBOSS online learning platform (https://www.amboss.com/de) and included written explanations, demonstration videos, and self-assessment quizzes. Students were given access to the online materials one week before the on-site course and were asked to complete all modules by then. The students were not reminded to complete the course. Face-to-face teaching time was reduced to a total of four hours and focused exclusively on hands-on scanning.

### Instructors

Both curricula were delivered exclusively by student peer tutors. To qualify, each tutor completed a four-week clinical ultrasound internship in the Ultrasound Department of Marburg University Hospital under expert supervision by a certified DEGUM level III specialist, the highest DEGUM qualification. In addition, all tutors received formal pedagogical training at the Dr. Reinfried Pohl Center for Medical Education. In both curricula each instructor supervised a maximum of four students, with a maximum course size of seven participants.

### Outcomes

The primary outcome was performance in an Objective Structured Clinical Examination (OSCE) [21] administered at the end of the curricula (maximum score: 20 points). The OSCE was specifically developed for this study in collaboration with the clinical head of the university hospital ultrasound unit in internal medicine and was aligned with the predefined learning objectives of the course. It assessed practical ultrasound competence, including probe handling, identification of anatomical structures, image interpretation, and diagnostic reasoning (supplementary file). Performance was evaluated using a predefined checklist with explicit scoring criteria and point allocation for each task (see supplementary file). Examiners were not permitted to intervene, provide feedback, or comment during the assessment. For logistical reasons, all tasks were performed at a single station. Each participant was given 8 min to complete all required tasks while examining healthy volunteer medical students.

Secondary outcomes included theoretical knowledge and learner perceptions (supplementary file). Theoretical knowledge was measured using a short written test. OSCE and written-test evaluators were not blinded to group allocation. In addition, students completed a questionnaire with five Likert-scale items addressing perceived overall knowledge gain, theoretical knowledge gain, practical skills gain, perceived course duration, and overall acceptance, complemented by free-text feedback.

### Thematic analysis of open-ended survey responses

Qualitative data from open-ended survey responses were analyzed using thematic analysis [22] supported by MAXQDA 2022. Two researchers (JK & FM) independently familiarized themselves with the dataset, generated initial codes inductively, and iteratively organized these codes into preliminary themes. Through ongoing comparison and discussion, themes were refined to capture shared patterns in students’ experiences and suggestions for course improvement. Coding discrepancies were resolved through consensus. Themes were then reviewed against the full dataset to ensure coherence and representativeness.

### Statistical analysis

A non-inferiority design [23] was applied using all randomized participants. The primary endpoint was the OSCE score. The null hypothesis stated that the blended-learning curriculum would be inferior to the conventional curriculum by more than the predefined non-inferiority margin (Δ = 4 points on the OSCE). The alternative hypothesis was that the blended-learning curriculum would be non-inferior to the conventional curriculum, with the between-group difference lying within this margin. The non-inferiority margin was set to Δ = 4. Sample size planning was based on the primary endpoint. Based on an expected standard deviation of 5 points and two-sided alpha of 0.05, at least 26 students per group were required based on a classical two-sample t-test assuming a difference of Δ to achieve a power of 80%. To account for potential dropouts and incomplete assessments, recruitment aimed for at least 35 students per group. Non-inferiority of the blended learning approach was assessed using a two-sided 95% confidence interval based on the corresponding t-distribution of the mean difference between the two groups. Non-inferiority can only be shown, if the confidence interval is completely covered by the non-inferiority margin. Secondary outcomes were analyzed as follows in an exploratory fashion. Theoretical knowledge test scores were summarized as means and standard deviations and compared between groups using independent two-sample t tests (two-sided with alpha 0.05). Course evaluations were analyzed descriptively using frequencies, percentages, means, and standard deviations as appropriate. All analyses were conducted in R, IBM SPSS Statistics, and Python (version 3.11.8) with the libraries pandas (version 1.5.3), NumPy (version 1.24.0), SciPy (version 1.14.1), and statsmodels (version 0.13.5).

## Results

**Fig. 1:**
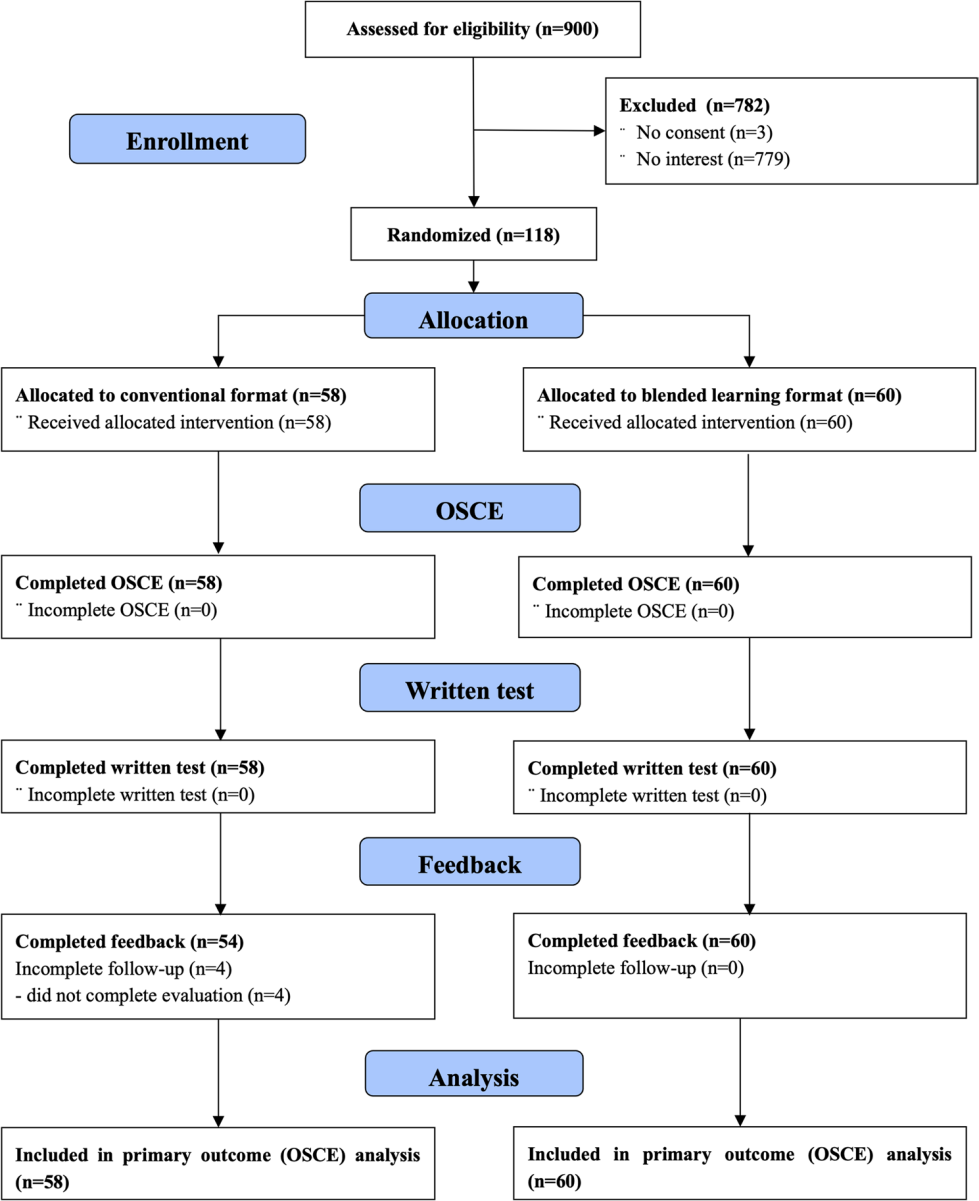
Trial participant flow diagram

Participants were recruited between October 29th 2022 and January 28th 2023. Of 121 students enrolled in the course, 118 provided consent and were included in the study. Sixty were randomized to the blended-learning curriculum and 58 to the conventional face-to-face curriculum (Fig. 1).

### Primary outcome

**Fig. 2:**
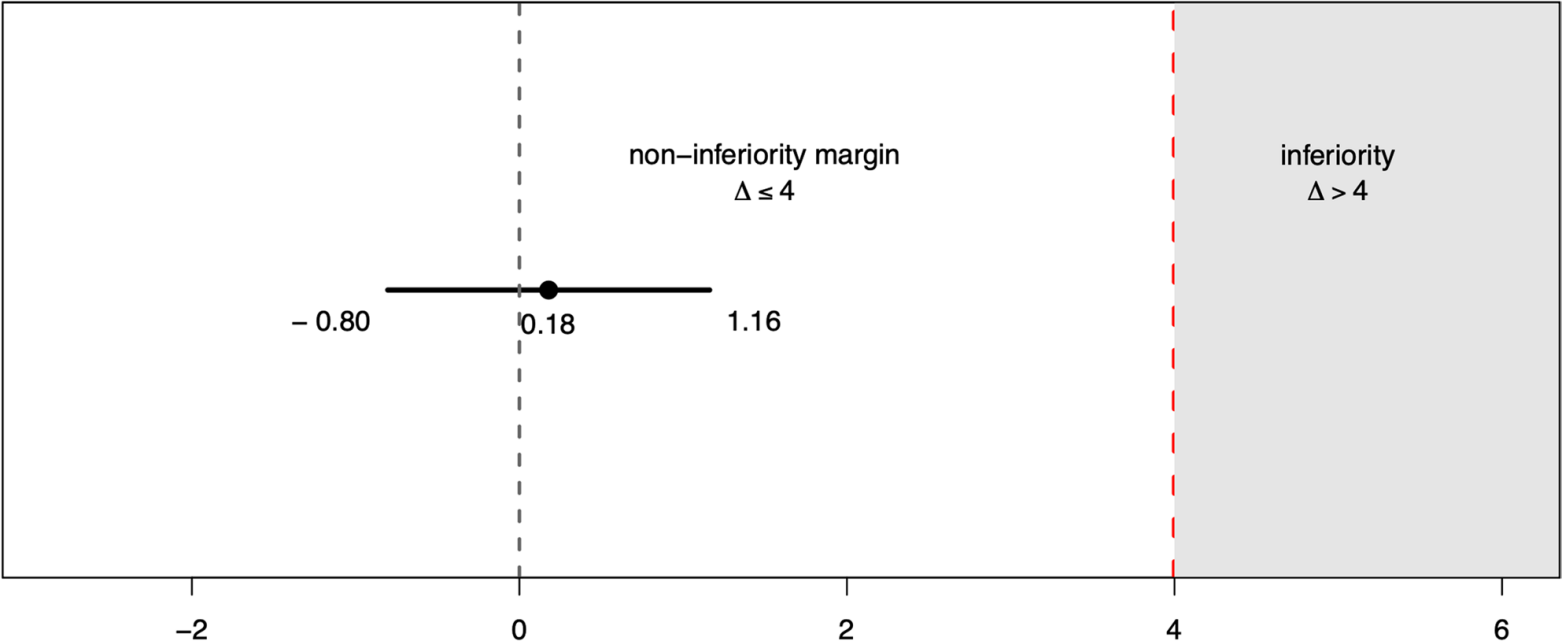
Non-inferiority plot of mean (95% CI) OSCE scores for blended learning versus conventional face-to-face curriculum

Between-group analysis indicated that blended learning achieved non-inferior OSCE total scores compared with the conventional format (14.68 ± 2.72 vs. 14.86 ± 2.67). The mean difference was 0.18 points (95% CI − 0.80 to 1.16), and the entire confidence interval lay within the predefined equivalence margin (Δ = 4), confirming non-inferiority of the blended learning approach (Fig. 2).

**Fig. 3:**
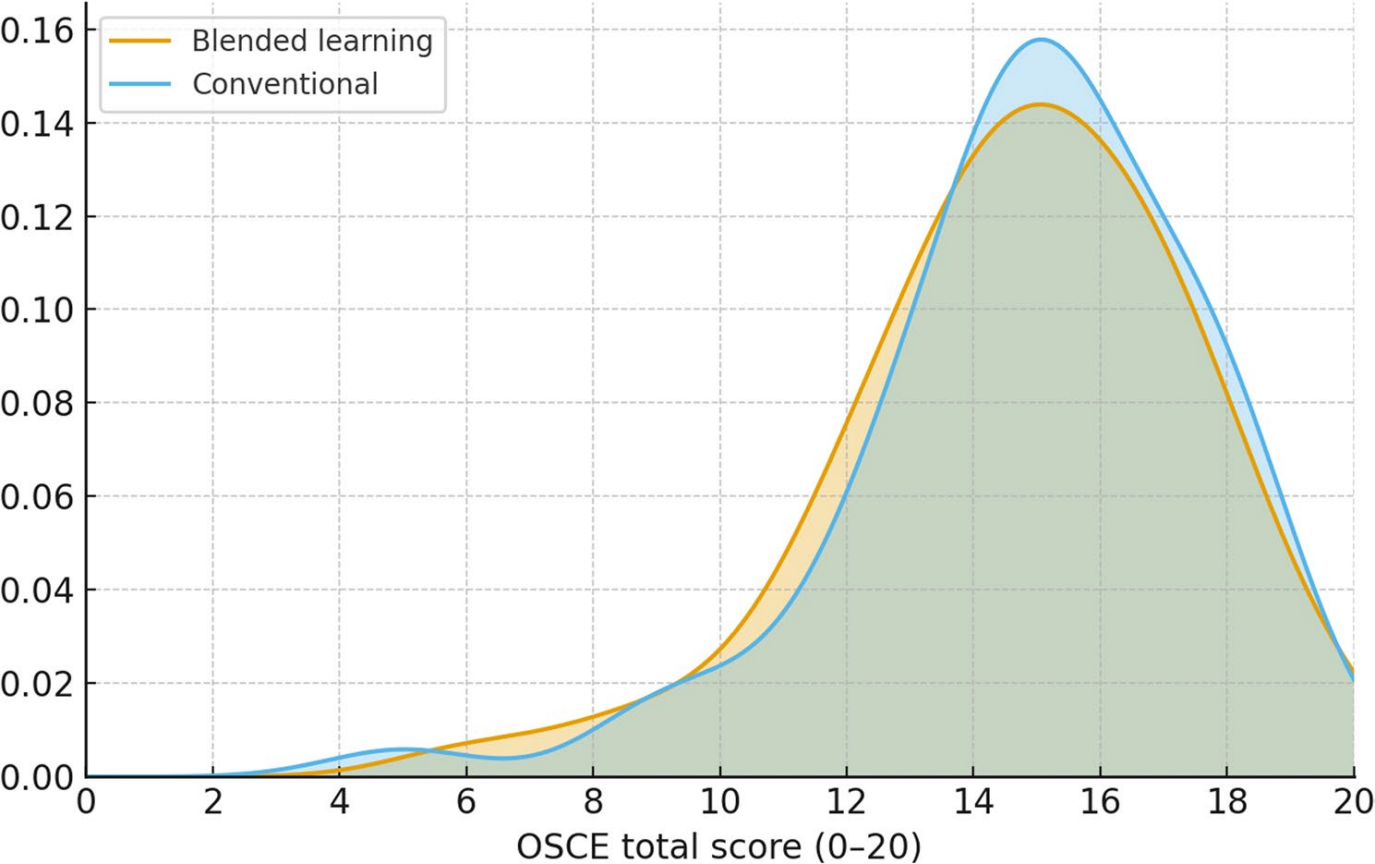
Kernel density plots illustrating distributions of OSCE total score (0–20 scale; 0 = minimum, 20 = maximum)

OSCE total scores in both groups showed slightly skewed, approximately normal distributions, with few participants achieving very low or perfect scores (Fig. 3). The only OSCE domain showing a significant between-group difference was device handling, which favored the conventional group (*p* = 0.04), see Table 2.

**Table 2:** Selected standard sections and respective mean scores

Test domain	Range of points	Mean score conventional curriculum (*n* = 58)	Mean score blended learning curriculum (*n* = 60)	*p*-value
OSCE				
Device handling	0–1	0.72	0.57	0.04
Kidneys	0–2	1.90	1.92	0.37
Liver	0–1	4.03	4.28	0.08
Gallbladder	0–1	0.66	0.80	0.05
Aorta	0–1	0.90	0.78	0.05
Spleen	0–2	1.45	1.23	0.06
FAST-protocol	0–8	5.21	5.10	0.32
OSCE total	0–20	14.86	14.68	0.36
Theoretical knowledge				
Cyst	0–2	1.81	1.33	< 0.01
Gallbladder	0–1	0.78	0.88	0.06
Spleen	0–1	0.93	0.90	0.27
Pancreas	0–1	0.69	0.70	0.45
Kidneys	0–1	0.90	0.92	0.36
Liver	0–1	0.64	0.63	0.48
Gallbladder	0–1	0.48	0.48	0.50
FAST goals	0–2	1.78	1.80	0.38
FAST sections	0–10	8.59	8.70	0.37
Theoretical knowledge total	0–20	16.59	16.35	0.35

### Secondary outcome

**Fig. 4:**
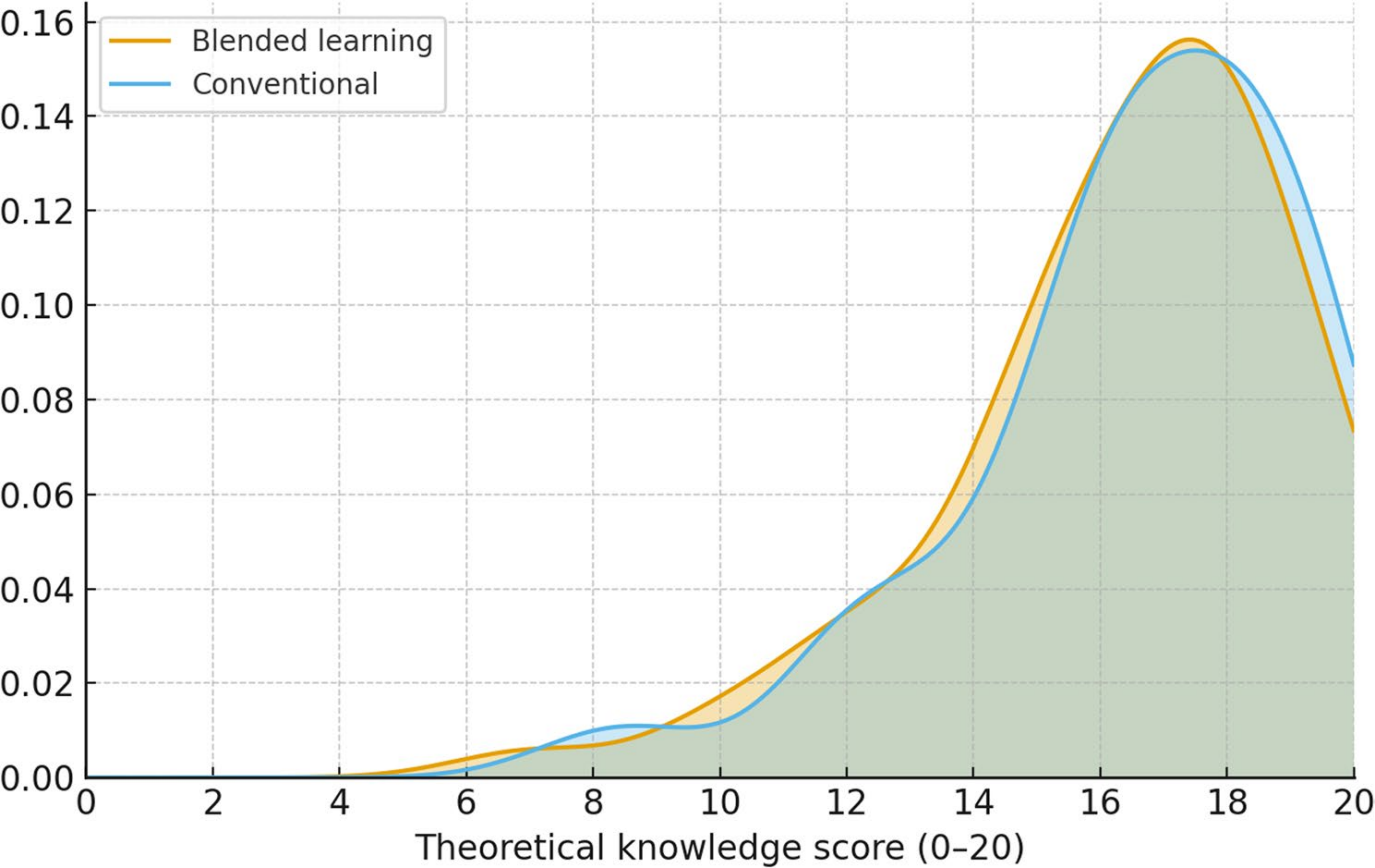
Kernel density plots illustrating distributions of theoretical knowledge score total score (0–20 scale; 0 = minimum, 20 = maximum)

The mean total theoretical knowledge scores did not differ significantly between groups (conventional: 16.59 ± 2.69; blended learning: 16.35 ± 2.74; *p* = 0.35), see Table 2. A significant between-group difference emerged only for the item “kidney cyst,” which favored the conventional group (*p* < 0.01). Total test scores in both groups again followed comparable right-skewed, approximately normal distributions (Fig. 4).

### Other outcomes

Perceived overall knowledge gain was high in both groups, with mean scores of 3.65 (conventional) and 3.53 (blended learning) on a 4-point scale. Theoretical knowledge gain was rated slightly higher in the conventional group (3.24/4 vs. 2.87/4), whereas perceived practical skills gain was nearly identical (3.61/4 vs. 3.67/4).

Course organization was rated as “good” or “very good” by 100% of conventional participants and 96% of blended-learning participants. In the conventional course, 85.2% rated the course duration as ‘just right’. 3.7% found the course ‘too short’ and 11.1% found it ‘too long’ or ‘much too long’. In the blended learning course, 91.7% of students rated the course duration as ‘just right’, while 8.3% rated it as ‘too short’ or ‘much too short’. None of the participants rated the course duration as ‘too long’ or ‘much too long’. In the conventional curriculum, all participants rated the course as ‘good’ (14.8%) or ‘very good’ (85.2%). The blended learning curriculum was rated as ‘poor’ by 3.4%, “good” by 20.3% and ‘very good’ by 76.3%.

### Open-ended constructive feedback

The results of the thematic analysis revealed several overarching themes related to instructional design, learning materials, clinical relevance, and course structure. Students in the conventional course proposed several improvements, including incorporating live ultrasound demonstrations into the theoretical segments, integrating theory more closely with practical scanning, and providing a digital script contrasting normal and pathological findings. They expressed a preference for fewer physical basics and more clinically relevant pathological cases. Additionally, participants suggested extending the course across two days and integrating it into the regular curriculum instead of scheduling it on weekends.

Students in the blended-learning group proposed extending the course duration to provide additional practice opportunities and expanding the anatomical scope to include the neck, heart, leg veins, and pelvic organs. They emphasized the need for pathological examples, given that peer scanning mostly reveals normal findings, and suggested integrating illustrative cases and providing a written course summary. Participants also recommended a brief review of the online theoretical content at the beginning of the session, as well as offering an optional tutor-led theory module for those preferring structured instruction.

## Discussion

This study examined whether the theoretical component of an extracurricular abdomen ultrasound course could be replaced with digital self-learning materials within a blended-learning format. The blended-learning approach demonstrated non-inferior performance in both practical OSCE outcomes and theoretical knowledge compared with the traditional course, while substantially reducing the need for on-site teaching to 4 instead of 8 h. These findings demonstrate that shifting theoretical content to digital self-directed learning can preserve educational effectiveness while enhancing student satisfaction, flexibility, and the efficient use of teaching resources.

These findings align with recent meta-analyses supporting the effectiveness of blended learning [24–26]. A recent study similarly showed that a blended-learning approach was effective for teaching bedside ultrasonography in Pulmonary and Critical Care Medicine fellowship programs [14]. These encouraging findings are especially relevant given that only about 56% of European medical curricula include hands-on ultrasound training, with an average of just seven hours of practical instruction per student [9]. In Germany, most medical faculties regard ultrasound education as important, yet consistently report shortages of time, personnel, and equipment [27]. Considering the growing demand for ultrasound training and limited teaching capacity, scalable and flexible approaches such as blended learning are urgently needed. Although the blended learning format reduces the bottleneck of tutor face-to-face time, it does not eliminate it, and access to ultrasound machines remains a limiting factor. Technological advances have already made handheld ultrasound devices more affordable, portable, and user friendly. The COVID-19 pandemic further accelerated interest in remote instruction, and studies such as the TELUS [28] and TELMUS [29] trials showed that remotely supervised abdominal and musculoskeletal ultrasound training can be as effective as traditional on-campus teaching. Simulator-based training offers standardized, around-the-clock hands-on practice and can enhance both theoretical understanding and practical scanning skills [30]. In addition, artificial intelligence is increasingly capable of providing real-time feedback on probe positioning and image quality, offering another avenue to expand access to high-quality ultrasound training [31].

Most students preferred a stronger emphasis on clinical and pathological examples. Many requested the inclusion of more pathological cases, which could be achieved using digital image libraries or simulation tools such as ultrasound simulators or virtual reality systems [32, 33]. Incorporating case-based teaching may further strengthen clinical reasoning. Evidence shows that case-based ultrasound instruction improves knowledge acquisition and learner engagement more effectively than traditional lecture-based formats [34].

Importantly, students in the blended-learning curriculum scored slightly lower in the OSCE subdomain of probe handling. This may reflect fewer opportunities for immediate tutor feedback on ergonomics and probe manipulation, as theoretical instruction in the blended format was shifted to self-directed online preparation rather than interleaved with hands-on practice. To mitigate this, future iterations could include a focused preparatory quiz on probe handling and machine settings with links to demonstration videos. Ultimately, probe handling is best acquired through repeated practice with real-time feedback, which can be provided through face-to-face teaching, tele-supervised instruction, or emerging AI-based guidance systems.

This study has several limitations. Students’ prior ultrasound experience was not formally assessed, although randomization likely minimized group differences. Basic demographic variables such as age, gender, or prior clinical exposure were also not collected, which limits the ability to explore subgroup effects or identify potential confounders. No long-term follow-up was conducted, leaving the durability of acquired skills uncertain. The single-center design may restrict generalizability to other institutions or curricular settings. Because neither participants nor instructors were blinded, performance or expectation bias cannot be fully excluded, despite blinded outcome assessment. Additionally, the OSCE and knowledge test were based on established standards but used scoring systems defined by the study team, similar to the non-inferiority margin. Real-life diagnostic performance was not assessed and could be addressed in future studies using as a more clinically relevant and ambitious outcome measure. Given the very small observed group differences, one could argue that the prespecified non-inferiority margin was relatively large and a smaller margin might also have been justifiable. The study was not pre-registered in a trial registry. Despite these limitations, the randomized design, adequate sample size, and use of objective performance measures provide strong evidence that blended learning can effectively support the acquisition of practical ultrasound skills. Moreover, the integration of the AMBOSS platform, which is widely used among German medical students, enhances the transferability of this approach to other faculties in Germany and strengthens the practical applicability of our results.

## Conclusions

This randomized controlled trial demonstrated that a blended-learning curriculum, in which theoretical content was shifted from face-to-face instruction to structured digital self-learning, was non-inferior to a traditional full-day ultrasound course for teaching practical abdominal ultrasound skills. Both approaches resulted in comparable OSCE performance, similar levels of theoretical knowledge, and high student satisfaction. By reducing on-site teaching time and relying on well-designed digital preparation, the blended-learning format provided a scalable and resource-efficient solution that helped address the persistent bottlenecks of limited personnel and equipment in ultrasound education. Overall, these findings support the integration of blended-learning strategies into undergraduate medical training and highlight their potential to expand access to high-quality, skills-oriented ultrasound training.

## Supplementary Information

Supplementary Material 1.

## Data Availability

The raw data supporting the conclusions of this article will be made available by the authors upon reasonable request.
