# Supplementary material for: Blended-learning with half the face-to-face time versus conventional abdominal ultrasound training in undergraduate medical education: a randomized controlled non-inferiority trial
Source: BMC Med Educ. 2026 Feb 28;26:561. doi: 10.1186/s12909-026-08914-4 (PMC13059292; doi:10.1186/s12909-026-08914-4)
Supplement: Supplementary file 1 — Supplementary Material 1. [file 12909_2026_8914_MOESM1_ESM.docx]

**OSCE form**

OSCE assessment form for sonography

Student pseudonym: _________________________

| Area | Task | Points |
| --- | --- | --- |
| **Ultrasound device & operation** | Can set focus, depth, etc. independently and appropriately & selects the correct transducer (tell the student) | _____ / 1 |
| **Kidney** | Display of a kidney in longitudinal and cross-section / 2 planes (1 point each) | _____ / 2 |
| **Liver** | Show 3 standard sections (1)  Show structures:   - Hepatic vein window + opening into v. cava (1) - Liver tip (1)   Echogenicity:   - Assess homogeneity and echogenicity of the liver (1) - Compare with echogenicity of the renal parenchyma (1) | _____ / 5 |
| **Gallbladder & bile ducts** | Correct visualization of the gallbladder  & exclusion of acute bile | _____ / 1 |
| **Abdominal vessels** | 2 standard sections for abdominal aorta | _____ / 1 |
| **Spleen** | Measurement in 2 planes (1)  At widest point (hilum section) (1) | _____ / 2 |
| **FAST protocol** | Depiction of (1 point each)  o Koller's pouch  o Morrison's pouch  o Douglas space  o Pericardial sac  o 2x costodiaphragmatic recess  In less than 1 min (2 points) | _____ / 8 |
| **Total** | | **_____ / 20** |

**Written test**

Pseudonym: ______________

A **cyst** usually appears on ultrasound as ______________ (echogenicity), with _______________________________________ (ultrasound phenomenon).

A typical sonographic sign of **cholecystitis** is _______________________.

The normal size of the **spleen** is approx. ____ x ____ x ____ cm.

In the transverse upper abdominal section, the **pancreas** runs in the above the __________________ (vessel).

A hypoechoic enlargement of the **renal** pelvis indicates _______________________.

If the **liver** is significantly more echogenic than the renal parenchyma, this is referred to as _______________________________________.

The **gallbladder wall** should be no more than _______ thick.

In **emergency sonography**, the aim is to rule out ___________________________________ and __________________________________.

Please complete the following table for the **FAST** scheme:

| Section / organ | Structures to be examined |
| --- | --- |
|  |  |
|  |  |
|  |  |
|  |  |

**Feedback**

**Questionnaire on subjective knowledge gain and satisfaction**

**for the Maris ultrasound course**

**Pseudonym: _____________________________**

1. How would you rate your **overall** knowledge gain from the ultrasound training?

| - Not at all | - Rather low | - Rather high | - Very high |
| --- | --- | --- | --- |
|  |  |  |  |

1. How would you rate your increase in **theoretical knowledge** from the ultrasound training?

| - Not at all | - Rather low | - Rather high | - Very high |
| --- | --- | --- | --- |

1. How would you rate your increase in **practical skills** from the ultrasound training?

| - Not at all | - Rather low | - Rather high | - Very high |
| --- | --- | --- | --- |

1. How would you rate the **duration** of the course?

| - Too short | - Rather too short | - Just right | - Rather too long | - Too long |
| --- | --- | --- | --- | --- |

1. How satisfied are you with the overall **course structure**?

| - Dissatisfied | - Rather dissatisfied | - Rather satisfied | - Satisfied |
| --- | --- | --- | --- |

1. What did you **particularly like** about the course? Regarding both contents and course structure.

|  |
| --- |

1. What could be **improved** in the course? Regarding both contents and organization.

|  |
| --- |
